# Atlanta Board of Health

**Published:** 1868-02

**Authors:** J. N. Simmons

**Affiliations:** Chairman Board of Health


### ATLANTA BOARD OF HEALTH.

### MORTUARY REPORT.

A comparative exhibit of the mortality of the city for the years 1866 and 1867, to the fourth quarter, shows that the number of deaths in 1867 is 100 per cent. less than in 1866, as will be seen by the subjoined statistical record:

| 1866. | DEATHS. | |
|---|---|---|
| | Whites. | Col'd. |
| 1st Quarter | 82 | 135 |
| 2d Quarter | 63 | 86 |
| 3d Quarter | 102 | 98 |
| 4th Quarter | 64 | 60 |
| | 311 | 379 |

| 1867. | DEATHS. | |
|---|---|---|
| | Whites. | Col'd. |
| 1st Quarter | 36 | 47 |
| 2d Quarter | 38 | 55 |
| 3d Quarter | 81 | 81 |
| | 155 | 180 |

This tabular statement also shows that the mortality among the Freedmen in 1866 and 1867, exceeds that among the whites, although the population of the latter, according to the last census, exceeds that of the former by 1,652.

Although considerable sickness of the remittent and intermittent types have prevailed the past season, especially among the destitute poor of the suburban portion of the city, yet we are assured that no other city can furnish a mortuary report indicating a locality more favorable to general health than the city of Atlanta.

J. N. Simmons,
Chairman Board of Health.

